# A qualitative study of professional caregivers' perceptions of processes contributing to mealtime agitation in persons with dementia in nursing home wards and strategies to attain calmness

**DOI:** 10.1002/nop2.24

**Published:** 2015-09-09

**Authors:** Ådel Bergland, Hilde Johansen, Gerd Sylvi Sellevold

**Affiliations:** ^1^ Lovisenberg Diaconal University CollegeOsloNorway; ^2^ Diakonhjemmet University CollegeOsloNorway

**Keywords:** Agitation, dementia, mealtime, nursing homes, professional caregivers' perspective

## Abstract

**Aim:**

Describe professional caregivers' perceptions of factors and processes contributing to mealtime agitation and strategies for attaining and maintaining calm mealtimes.

**Design:**

Qualitative and descriptive.

**Methods:**

A convenience sample of professional caregivers working in two wards for residents with dementia was used. Data were collected during two focus‐group interviews and supplemented with field notes from six reflection groups. Thematic content analysis was conducted. Data collection occurred from 2010–2011.

**Results:**

Professional caregivers perceived agitation during mealtime as resulting from negative feelings in residents triggered by a lack of or negative social interaction, too much or ambiguous stimuli or demands exceeding residents' capacity. Strategies for attaining calm mealtimes involved thorough planning beforehand. During mealtime, professional caregivers focused on establishing a positive community around the table, helping residents focus on eating and continuously observing residents for subtle signals indicating that agitation was about to develop. The prerequisites to succeed with the strategies were knowledge of the residents' preferences and abilities, knowledge sharing within the team and awareness of one's own communication style. Thus, the professional caregivers operationalized person‐centred care in a mealtime context.

## Introduction

### Background

As the disease process of dementia progresses, persons with dementia need increasing assistance with daily tasks including eating (Watson & Green 2006, Lin *et al*. 2010, Jackson *et al*. 2011). Various types of nutritional difficulties can occur in advanced stages of dementia diseases and consequently, nursing home residents with dementia are at risk of weight loss and malnutrition (Amella 2004, Chang & Roberts 2008, Lin *et al*. 2010). Eating difficulties involve not being able to move food from the plate into the mouth and chew and swallow it and are usually caused by disease processes such as difficulties understanding what a meal is, using a knife, using a fork and/or spoon or forgetting how to swallow once they get the bolus of food in their mouth (Chang & Roberts 2008, Aselage *et al*. 2011, Jackson *et al*. 2011). Feeding difficulties refer to the interaction between persons with dementia and persons assisting them with eating. Persons with dementia can show aversive behaviour during feeding such as keeping their mouth shut, turning their head away and spitting out food (Pasman *et al*. 2003). Pasman *et al*. (2003) reported that nurses reacted differently to aversive behaviour and that their interpretations of aversive behaviour during feeding were crucial and decisive regarding whether the resident received more food or if the nurses stopped feeding.

A concept analysis conducted by Aselage and Amella (2010) described mealtime difficulties as a broader concept than eating and feeding difficulties and with the following attributes: mealtime patterns, dyad interaction, mealtime environment, dementia and aversive feeding behaviours (p. 34). Liu *et al*. (2014) summarized the current literature and stated that mealtime difficulties in persons with dementia include ‘any difficulty or problem occurred during eating, feeding or mealtime associated with physical, cognitive, behavioural, social, environmental and cultural factors’ (p. 15).

A specific type of mealtime difficulty is mealtime agitation, which is when residents have inappropriate verbal, vocal or motor activity not explained by needs that are evident to the observer (Cohen‐Mansfield *et al*. 1989, Gilmore‐Bykovskyi 2015). Examples of mealtime agitation can be shouting or screaming, asking questions constantly, being aggressive, resisting eating, being restless, or wandering, including leaving the table before the meal is finished (Cohen‐Mansfield *et al*. 1989, Denney 1997, Gilmore‐Bykovskyi 2015). Not understanding the ‘meal situation’ or not understanding the help offered by staff can result in various types of agitated behaviours during mealtime (Aselage & Amella 2010).

Although general agitation and behaviour difficulties among persons with dementia diseases have been extensively explored (Zuidema *et al*. 2007, Cohen‐Mansfield *et al*. 2012, Selbaek & Engedal 2012), few studies have investigated behavioural difficulties specifically during mealtime. However, Zuidema *et al*. (2007) reported a correlation between aberrant motor behaviour, night‐time behaviour and eating disorders among patients with severe dementia. Additionally, White *et al*. (2004) found that residents with low BMI had higher frequency and severity of behavioural problems and concluded that behavioural disturbances play a role in weight problems among persons with Alzheimer's disease. Wandering nursing home residents may have difficulties sitting down at the table long enough to eat sufficiently (Beattie *et al*. 2004). Studies exploring interventions to reduce agitation during mealtime are scarce. These studies mainly describe various ways of using music to prevent agitated behaviour and to create a quiet and positive mealtime atmosphere (Richeson & Neill 2004, Hicks‐Moore 2005, Johnson *et al*. 2011). A study also reported that giving information using a clock and a sign with information about when the meals started reduced questions and agitation before meals (Nolan & Mathews 2004). Studies that focus on assisting residents with dementia during mealtime describe strategies to prevent agitation between one caregiver and one resident (Mamhidir *et al*. 2007, Aselage & Amella 2010, Liu *et al*. 2014) but not in groups of residents.

Several essential factors are known to contribute to a positive mealtime setting and result in increased food and liquid intake and improved nutritional status. These factors include a homelike environment with small and family‐like groups and dining rooms (Jackson *et al*. 2011), including serving food in a homelike manner, giving residents an opportunity to choose food and quantity for consumption and avoiding pre‐plated service trays (Jackson *et al*. 2011, Liu *et al*. 2014). Further evidence suggests that soothing music, high contrast tableware, personalized food and individual feeding assistance are essential for making a positive and appropriate mealtime setting for persons with dementia (Jackson *et al*. 2011, Liu *et al*. 2014). Last, it is also very important to provide integrity‐promoting care (Mamhidir *et al*. 2007, Liu *et al*. 2014).

However, we have not been able to identify any study describing professional caregivers' (PC) perceptions of factors and processes that lead to mealtime agitation and which strategies PCs perceive as most effective for attaining a calm and quiet mealtime atmosphere.

### Aim

This study is part of a larger action‐research study (Hummelvoll & Severinsson 2005) that aims to develop the quality of nursing home care and facilitate the development of competence among PCs.

The aims of the study reported in this article are to (1) describe PCs' perceptions of factors and processes contributing to mealtime agitation in nursing home wards for people with dementia and (2) describe PCs' strategies for attaining and maintaining calm mealtimes.

In this study, PCs include Registered Nurses (RN), State Enrolled Nurses (SEN), Nursing Assistants (NA) and Nursing students in the third year of their bachelor education. Norwegian State Enrolled Nurses have two years training at an upper secondary level and nursing assistants have no formal training. These groups work under supervision from a RN.

## The study

### Design

The design was qualitative and descriptive.

### Setting and sample

The study was conducted in a 127‐bed nursing home in a large town in Norway. The nursing home is composed of six wards, two of which were included in the study. These wards had residents with dementia and were selected by the unit nursing officer because their head nurses had reported challenges related to mealtime agitation. The participants in the study were recruited among PCs from the two wards and third year bachelor nursing students who had clinical studies in these wards.

### Data collection

Data were collected during two focus‐group interviews and six reflection groups. A group of registered nurses and state enrolled nurses participated in focus‐group interviews in the first phase of this study. The purpose of these interviews was to explore the PCs' experiences and understandings of mealtime agitation and calm mealtimes by using the group dynamics where the PCs stimulated each other by responding to others' statements (Polit & Beck 2012). The main inclusion criterion was that the participants were deemed by their colleagues and the head nurse to be good at contributing to a calm atmosphere while on duty and thereby being role‐models in the ward. Before the focus‐group interviews, all participants were asked to write down a history describing a mealtime with substantial noise, restlessness and agitation among residents and they were asked to describe how they turned this situation into a calm mealtime. They were asked to describe the event as concretely as possible. These histories were taken as a starting point in the focus‐group interviews. During the interviews, the participants discussed further and more thoroughly how they perceived mealtime agitation and how they worked to attain a calm mealtime. Examples of interview questions are listed in Table 1. Data from the focus‐group interviews were the main source in this study.

**Table 1:** Questions asked during focus‐group interviews and reflection groups

Data collection method	Questions asked
Histories written before focus‐group interview	Describe an episode of agitation during mealtime and how you managed to ‘turn the situation’ into a calm mealtime Provide a written example describing how you work to prevent episodes of agitation from developing.
Focus‐group interviews	Is there something from the written stories you want to elaborate? What do you see as the most important factors contributing to calmness during meals? How do you work to attain a calm and quiet mealtime? How do you prepare the residents for mealtime? How do you perceive the PCs' role during mealtime?
Reflection groups	Has anyone had an episode we can discuss today? What happened in this situation? How did the residents react to what happened during the mealtime? What were you doing? What does agitation mean? What does calmness mean? You often talk about residents who are shouting. How can we interpret shouting? Can you describe what you were doing when the residents sat calmly at the table and enjoyed the meal? How can you change a mealtime with agitation into a calm mealtime? How can agitation during mealtime be prevented?

In the second phase of the study, during a 6‐month period following the focus‐group interviews, six reflection groups were conducted. The main purpose of these reflection groups was educational by giving the participants opportunities to explore concrete episodes thoroughly and thereby increase their understanding of what happened during incidents of agitation and also to share knowledge that the PCs already possessed. The discussions revealed the PCs' perceptions of mealtime agitation and mealtime calmness that supplemented data from the interviews. The focus in these group sessions was on agitated and calm mealtimes and strategies for changing a noisy and agitated mealtime into a calm one. The group sessions were initiated by asking one of the participants to describe a specific episode with agitation during a meal, usually a recent episode. The leader encouraged the participant to describe in detail what happened and also to reflect on why this episode resulted in agitation and which actions contributed to reducing the agitation and attaining a calm mealtime. Examples of the questions asked are reported in Table 1. Registered Nurses, state enrolled nurses, nursing assistants and third year nursing bachelor students participated in these reflection group sessions. Those who were on duty when the reflection group was scheduled participated and thus, the attendees of each reflection group varied. Data from the reflection groups were used to supplement the data from the focus‐group interviews.

Both the focus‐group interviews and the reflection groups lasted approximately 60 minutes and were led by one of the researchers (GSS), with the exception of one reflection group that was led by HJ. During both the focus‐group interviews and the reflection groups, one of the researchers (ÅB) took extensive and detailed notes from the discussions. Immediately after each session, the notes were reviewed and details from the discussions were completed. The reason for not using a recorder was due to concern that the presence of a recorder, which was an unfamiliar piece of equipment for many of the participants, could result in them presenting fewer or less thorough reflections. The study lasted for 12 months. Data were collected from 2010–2011.

### Analysis

The PCs' – e.g. the role‐models' – written histories and the texts from both the focus‐group interviews and the reflection groups were analysed as a whole. However, the texts from the focus‐group interviews were analysed first and the content from the histories and the reflection groups were used to supplement the descriptions of the themes. We conducted a content analysis inspired by Malterud's (2012) systematic text condensation. First, the whole interview text was read several times to obtain a first impression and to identify preliminary themes. The text was read with the following questions as a starting point: How did the PC understand episodes of agitation? Which factors were perceived as contributing to agitation? How did the PCs describe their own way of interacting with residents during meals? How did they work to create peacefulness? How did the PCs describe the process of transforming an agitated mealtime into a calm one? Initially, the PC's role and communication style, knowledge about the residents and meal planning were identified as preliminary themes and corresponding meaning units were identified, coded and organized. During this part of the analysis process, the text was read several times and the preliminary themes were further developed and refined into the following main themes: factors and processes contributing to mealtime agitation, strategies for attaining a calm mealtime atmosphere and prerequisites for succeeding with the strategies. Then, the whole text was read again and text‐units related to these themes were identified, reorganized and read several times to explore and describe the content of each main theme and how they were related. The texts were read and analysed both individually and in the group meetings of the research team. The results were also discussed with some of the PCs from the participating wards.

### Ethics

The study was assessed by the Norwegian Social Science Data Services (NSD) as not involving sensitive data because the conversations with the participants were not tape‐recorded. The participants gave their written consent prior to focus‐group interviews and reflection groups. All episodes involving residents discussed during the focus‐group interviews and reflection groups were anonymized.

## Results

### Characteristics of the participants

Twenty‐four PCs (21 women and 3 men) participated in the study. The largest groups were state enrolled nurses (*n *=* *8) and last year nursing students (*n* = 8) and three of the participants were Registered Nurses (RNs). The participants' work experience in nursing homes varied considerably. Work experience in the specific wards included in the study varied from 1·5 months to 7 years (See Table 2).

**Table 2:** Sample characteristics (*N *=* *24)

Characteristics	*N*
Profession	
Registered nurses	3
State enrolled nurses	8
Nursing assistants	4
Nursing students (third year)	8
Others	1
Gender	
Female	21
Male	3
Age	
20–40 years	12
41–60 years	11
Missing	1
Work experience in nursing homes^a^	
1 month–4 years	7
5 years–10 years	4
More than 11 years	4
Work experience in the ward^a^	
1 month–4 years	10
More than 5 years	5

a Nursing students not included (*N *=* *15).

### Overview of major findings

The PCs described mealtime as an important event in the wards for persons with dementia diseases. A calm mealtime was viewed as essential for ensuring that the residents were able to eat sufficiently and that mealtime was a positive experience for the residents. The PCs claimed that agitation could easily develop during mealtime. They described their own role as essential and acknowledged that their own behaviour could cause agitation. The PCs also viewed themselves as crucial to establishing a calm mealtime and preventing the development of agitation.

### Incidents resulting in negative feelings and development of agitation

**Figure 1:**
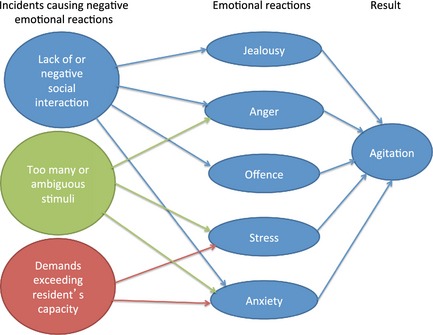
Caregivers' perceptions of processes contributing to mealtime agitation.

The PCs experienced that agitation during mealtimes frequently resulted from residents' negative emotional reactions and feelings, such as jealousy, anger, offence, stress and anxiety. Various types of incidents could result in these negative feelings, as illustrated in Figure 1.

*Lack of social interaction* around the table also caused jealousy and anxiety among the residents. For example, when the PCs assisted a resident with eating and focused their attention entirely on that person, other residents around the table or in the dining room would get jealous and envy the resident receiving the PC's attention. If family members were present and talked extensively with the PCs, their attention to the residents was often lost and the residents became jealous. Residents who were not involved in the community or in the conversation at the table would get *anxious* because they wondered if they were the topic of the conversation.

*Negative social interaction around the table* caused offence as well as anger. For example, when residents expressed critical or negative statements about other residents, arguments would easily develop. Additionally, if family members expressed negative comments about a resident, the resident(s) being spoken of usually reacted negatively to these statements and the PCs described them as being offended. Residents around the table who coughed or ate in an unappetizing manner made peer residents angry and these peers insisted that this resident should leave the table.

*Too many stimuli* in the ward resulted in stress, anxiety and anger among the residents. Both persons and activities could be distracting factors and also cause over‐stimulation. If too many persons were present in the dining room and some entered or left the room, it was difficult for the residents to concentrate on eating. If the persons left the dining room, some residents would experience this exit as a signal that they should also leave the table. Some residents even became anxious if a PC, they were especially attached, to left the room. Activities that took the focus away from eating, such as administering medication during mealtime, were also disruptive and upsetting for the residents. Additionally, if the water tap was turned on before the mealtime ended, some residents would interpret this action as the meal was finished and would become stressed if they had food left on their plate. For some residents, various sounds from the radio, TV and music, as well as phone ringing, were described as distracting and distressing noise stimuli. Even repeated questions or shouting from peer residents were experienced as noise stimuli and caused anger in some residents. Additionally, too much sound resulted in residents not hearing what other persons around the table were saying, which would interrupt the conversation and cause some residents to become irritated. As with auditory stimuli, visual stimuli through windows into the corridors were also distracting during mealtime.

*Unclear or ambiguous stimuli* also caused anxiety. Two examples described by the PCs were when residents were not able to understand and interpret sounds in the ward and identify where they came from and when darkness outside the window led to anxiety.

#### Demands exceeding the residents' abilities and capacity

Residents became stressed and anxious if they experienced situations they were unable to manage, such as being served food they were not familiar with and not recognizing or understanding how to eat. Residents with severe dementia also had difficulties in using cutlery. Conversations during meals were too demanding for some of the residents because they could not cope with eating and talking simultaneously.

### Strategies to attain and maintain a calm mealtime

**Figure 2:**
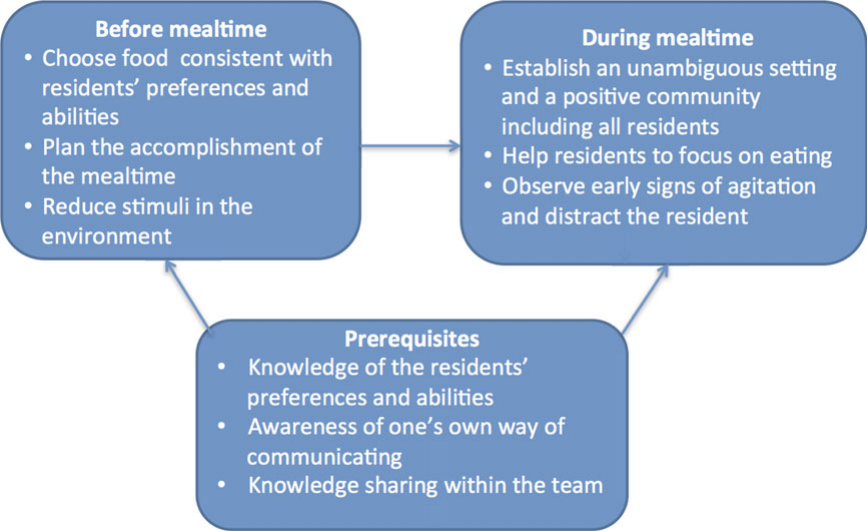
Strategies to attain and maintain a calm mealtime.

To create and maintain a calm mealtime, the PCs described strategies they used to plan and conduct the mealtime in ways that did not result in episodes with negative emotions in the residents. The PCs tried to arrange a mealtime where the residents could experience positive feelings (Figure 2).

### Strategies before the meal

The PCs emphasized that good planning was essential to a calm mealtime and that several actions were involved in the planning process.

#### Choosing food consistent with the residents' preferences and abilities

The PCs indicated that it was important to provide residents with food they knew and could eat without much difficulty. For some residents, this food was able to be eaten with a spoon or with their hands because eating with a knife and fork was too difficult. The PCs also emphasized the importance of the residents' ‘food traditions’ and serving food that was familiar to the residents. Food and the smell of food were often associated with positive memories that could trigger positive feelings in the residents.

#### Plan the course of the meal

Having a good plan for how the meal should be conducted and agreeing on which tasks and roles each PC would have during the mealtime were described as critical to achieving a calm mealtime. It was essential to decide in advance who would sit down at the table and who would serve the residents. The PCs also had to assess carefully when to set the table. It was important to set the table before the meal was to begin but not too long before, because some residents would expect the meal to begin as soon as the table was ready and could be upset by perceived delays. Another issue of importance was the timing of each resident's arrival at the table and this issue had to be assessed individually for each resident. Some residents could get to the table, wait for the food to be served and enjoy waiting while watching the PC prepare the meal, whereas other residents had a better experience if they arrived at the table just before the food was served and could start eating immediately. The order in which the residents were served also needed to be considered. Some residents had to get food immediately so that they did not get angry and leave the table, whereas others could wait as the other residents were served their food.

#### Reducing stimuli

An important part of the preparation was to reduce stimuli in the dining room. One method was to shut the door to the room. A closed door was a barrier which minimized the likelihood of persons entering and leaving the room and for those who did enter, it forced them to do so more cautiously. Additionally, a closed door minimized the sounds from activities and persons in other parts of the ward. Another way of reducing stimuli was to turn off the radio, TV and music. If the dining room had windows into the corridor, curtains or blinds helped to reduce visual stimuli. The PC also emphasized the importance of not administering medication during mealtime.

### Strategies during mealtime

*Establish an unambiguous setting and a positive community including all the persons around the table*. The PCs' presence during mealtime was described as essential for securing a safe, predictable and calm mealtime for the residents. The PCs also reported that when they sat at the table, the mealtime endurance was longer and the residents ate more. To establish a positive community, the PCs sitting at the table needed to focus continuously on the residents and ‘be present and involved’ in their interactions with the residents. An important task was to initiate conversations that included all the residents around the table. It was essential to choose topics that were experienced as positive by all the residents. PCs needed to use short sentences and have eye contact with the residents. PCs also had to observe whether some of the residents stopped eating because they were not able to eat and participate in a conversation simultaneously. To establish a positive community around the table, it was also important to have a clear beginning and ending to the mealtime (i.e. to frame the mealtime). It was important that the meal was completed simultaneously around each table and that the residents had similar food. The PCs reported that if one resident had the main course, another had dessert and the third was drinking coffee, agitation could easily develop because residents often focused on what the others were eating. If family members were present at the table, they also had to be included in the community. In the PCs' experience, if a positive community around a table was established, residents could tolerate more stimuli than was considered optimal.

#### Help the residents focus on eating

The PCs described the importance of helping each resident to maintain their attention on the food and on eating during the meal and assisting them if necessary. This task could be performed by sitting together with the residents and continuously encouraging them to eat and focus on their food and not on the others around the table or at other tables. These residents were often distracted by talk, which could cause them to stop eating. Another way of helping residents to maintain focus on eating was to set the table in a ‘simple way’ – by having only the necessary things on the table. Helping residents to focus on eating also implied placing the food in front of the resident and using plates with various colours to ensure that the resident could see the food clearly.

#### Observe early signs of agitation and divert residents' attention

The PCs needed to continuously observe the residents' body language and early signals or expressions indicating that agitation were about to develop. It was essential to know each resident's subtle signals. One female resident would wrinkle her nose in a particular manner, whereas other residents would get ‘shifty‐eyed’. When these signs were observed, it was necessary to divert the resident's attention immediately, which might involve changing the subject of the conversation or singing on some occasions. If the PCs were able to use humour in a way that the residents could understand, this method would also calm a situation where agitation was about to develop.

### Prerequisites to succeed with the strategies

The PCs perceived mealtimes as complex situations and they believed that particular prerequisites were critical to their success with the described strategies for achieving a calm mealtime and a good experience for residents. Knowledge was essential in the planning process as well as during the mealtime. Knowledge about each resident and his/her life story was important. This knowledge included information about each resident's food habits and their generation's food traditions and traditional dishes. Additionally, knowledge about which type of food each resident preferred and was able to eat and how the food should be served to make him/her eat was essential. One of the state enrolled nurses described a female resident who did not eat unless her slices of bread were cut in a very specific manner. Knowledge about each resident's diagnosis and how the symptoms affected the resident and his/her behaviour were important. Additionally, knowing the residents’ signs and signals indicating that agitation was about to develop was essential.

Another prerequisite described was the PCs’ awareness of their own way of communicating with the residents, especially their own body language and how these actions affected the residents. Many residents were good at reading the PCs’ body language and reacted if they interpreted it negatively. For example, the use of large gesticulations or rapid movements resulted in the residents getting anxious. One of the PCs described that when she first started, she waved her arms often during conversations around the table, but she noticed that some of the residents would become frightened and so she modified her body language to avoid causing this reaction. Last, sharing knowledge within the team was also described as an essential prerequisite for success in using these strategies.

## Discussion

A main finding in this study was that PCs perceived the development of agitation during mealtime as being caused by residents’ ‘negative’ emotional reactions, which were triggered by negative or inadequate interactions with PCs, peer residents or family members as well as environmental factors. The PCs also developed strategies for managing the mealtime, with the aim of triggering positive emotions among the residents. The PCs emphasized that the interaction and community between residents with dementia and between residents and other persons in the ward easily made residents vulnerable.

A physical and psychosocial environment that has been adjusted to the needs of the resident is crucial to achieve high quality dementia care (Bergland & Kirkevold 2011, Willemse *et al*. 2015). The PCs seemed to work in accordance with essential knowledge in dementia care by focusing on reducing stimuli, choosing food that residents were able to eat and arranging mealtimes by considering each resident's abilities and preferences. The PCs seemed to be aware of the importance of not giving the residents challenges that exceeded their capacity. The theory of Progressively Lowered Stress Threshold (Hall & Buckwalter 1987) argues that the stress threshold in persons with dementia is reduced as the disease process develops and their condition deteriorates. The PCs acted in accordance with this theory when they described agitation as resulting from over‐stimulation during mealtime and when they continuously looked for subtle signals indicating that the resident was about to cross their stress threshold and become agitated.

This study contributes to the research literature by describing PCs’ understanding of how mealtime agitation in persons with dementia can develop. By understanding mealtime agitation not only as a result of the dementia disease but also as a result of negative feelings resulting from interactions with other persons or person–environment interactions, the PCs considered the residents’ perspective and perceived agitation as a way of communicating that they were not comfortable. As a result, the PCs were able to take action and arrange for a calm mealtime. In describing in detail strategies of considering each resident's needs, the caregivers also described how mealtime might be planned and conducted according to the principles of person‐centred care (PCC).

The core aspects of PCC in dementia care are to acknowledge the personhood of persons with dementia in all aspects of care, to perceive the personhood as concealed rather than lost as the disease process progresses, to emphasize a respectful relationship and to prioritize relationships as much as care tasks, to collect and use personal life experiences to individualize the care and the environment and to offer shared decision making and interpret behaviour from the person's point of view (Edvardsson *et al*. 2008, Morgan & Yoder 2012). PCC is a concept that has been criticized for being abstract and lacking descriptions of what such care actually implies (Edvardsson *et al*. 2008, Morgan & Yoder 2012). Additionally, Rokstad (2013) stated that implementation of PCC is a complex and challenging task. In the present study, we argue that the PCs described how a mealtime can be conducted according to principles of PCC. They operationalized what PCC might involve in a mealtime context without specifically mentioning the concept. The PCs in our study perceived mealtime agitation as developing because the residents were reacting negatively to frustrating interactions or environmental factors and this reaction resulted in negative emotions such as anger, jealousy and stress. The PCs thereby interpreted agitation as the residents’ way of communicating unmet needs or that they felt uncomfortable. Accordingly, the PCs interpreted and understood the mealtime situation from the residents’ perspective. The PCs also emphasized the importance of meeting each resident in his/her situation and giving attention to their individual needs. The PCs individualized their care related to mealtime, as evident in their assertion that the ideal timing of each resident's arrival at the table had to be individually assessed. Another way of personalizing care was for PCs to offer food that each resident preferred, was familiar with and was able to eat. The PCs also prioritized relationships with the residents as much as tasks when they would spend time sitting at the table with the residents during mealtime. By emphasizing the development of a positive interaction and community around the table, they also described the development of a positive social environment including all the residents, which is another aspect of PCC (Morgan & Yoder 2012).

The PCs also experienced that when a mealtime was conducted according to the strategies they described, agitation usually did not occur. Whether PCC reduces agitation in persons with dementia in nursing homes has not yet been empirically established. Sjogren *et al*. (2013) reported no correlations between PCC and levels of agitation. However, Rokstad *et al*. (2013) reported that Dementia Care Mapping and VIPS, two approaches included in PCC (Brooker 2003), resulted in reduced levels of agitation as measured by the Neuropsychiatric Inventory Questionnaire (NPI‐Q) but not according to the Brief Agitation Rating Scale (BARS). Consequently, more research is needed to explore the relationship between PCC and agitation, including during mealtime.

### Study limitations

The study was conducted in one nursing home and could have benefited from including more institutions. The study could also have benefited from using multiple methods. By combining participant observation and interviews, the researchers would have been able to observe how the PCs acted before and during the meal and discussed these observations during interviews to explore their perceptions more thoroughly.

## Conclusion

This study reports nursing home PCs’ descriptions of how mealtime agitation develops and their strategies for attaining a calm mealtime for persons with dementia. By perceiving mealtime agitation as being caused by negative emotions that are triggered by distressing inter‐personal or person–environment interactions, they interpreted the situation from the residents’ perspective and were able to take actions and, in many situations, prevent episodes of agitation. In working to attain a calm mealtime, the PCs emphasized adequate planning before the mealtime and arranging for positive and inclusive interactions and a feeling of community around the table. To achieve this aim, knowledge of the residents’ preferences and abilities and awareness of one's own communication style were crucial.

### Clinical implications

When PCs perceived agitation as developing from negative emotional reactions to episodes that were happening during the meal, they are able to take actions to prevent these episodes. This approach is different from an attitude that perceives agitation as solely caused by the dementia disease and thus as something that the staff cannot influence or prevent.

Head nurses in nursing home wards for persons with dementia should be aware of the need for knowledge about how to conduct positive mealtimes and should ensure that PCs receive adequate support and supervision while participating in mealtimes. Head nurses should communicate to their staff that participating during mealtimes is an essential part of high quality dementia care.

## Author contributions

Study design: ÅB, GSS; data collection: ÅB, GSS, HJ; data‐analysis: ÅB, GSS, HJ and manuscript preparation: ÅB, GSS, HJ.

All authors have agreed on the final version and meet at least one of the following criteria [recommended by the ICMJE (http://www.icmje.org/recommendations/)]:

- substantial contributions to conception and design, acquisition of data, or analysis and interpretation of data;
- drafting the article or revising it critically for important intellectual content.
